# A Nationwide Cross-Sectional Survey of UK Breast Surgeons' Views on the Management of Ductal Carcinoma In Situ 

**DOI:** 10.1155/2015/104231

**Published:** 2015-11-30

**Authors:** Gurdeep S. Mannu, Joao H. Bettencourt-Silva, Farid Ahmed, Giles Cunnick

**Affiliations:** ^1^Oxford University Hospitals, NHS Trust, Oxford OX3 9DU, UK; ^2^Norfolk and Norwich University Hospital, Norwich NR4 7UY, UK; ^3^Buckinghamshire NHS Healthcare Trust, Buckinghamshire HP11 2TT, UK

## Abstract

*Background*. There is wide variation in the management of Ductal Carcinoma In Situ (DCIS) nationwide. We aimed to investigate whether the attitudes of surgeons towards different aspects of DCIS treatment varied by seniority of surgeon or by geographical region within the UK.* Materials and Methods*. A nationwide online survey targeted at UK breast surgeons was undertaken. The anonymous survey contained questions regarding demographics of respondents and specific questions regarding DCIS management that were identified as areas of uncertainty during a systematic search of the literature.* Results*. Responses from 80 surgeons were obtained. Approximately 57% were male and the majority were consultant or specialist registrar. Approximately 63% of participants were based in district general hospitals with all training deaneries represented. Surgeons' views on the prognosis and management of DCIS varied geographically across the UK and terminology for DCIS varied with surgeon seniority. Surgeons' views particularly differed from national guidance on indications for SLNB, tamoxifen, and follow-up practice.* Conclusion*. Our survey reaffirms that, irrespective of national guidelines and attempts at uniformity, there continues to be a wide variety of views amongst breast surgeons regarding the ideal management of DCIS. However, by quantifying this variation, it may be possible to take it into account when examining long-term trends in nationwide treatment data.

## 1. Introduction

Ductal Carcinoma In Situ (DCIS) is the most common type of noninvasive breast tumour and, since the introduction of breast screening, the number of women diagnosed with DCIS now accounts for approximately one-in-five tumours detected by the NHS screening programme [1]. However, there is little reliable information on the prognosis of DCIS at present. It is unclear which patients with DCIS would go on to develop invasive breast cancer (IBC) and over what period of time. Of those women who receive treatment, the aggressiveness of treatment for a condition, which lacks the capacity to spread, is contentious. In particular, the optimum surgery, margin thickness, and axillary surgery in the management of DCIS remain uncertain. In addition, the impact of adjuvant radiotherapy or endocrine therapy is not clear, nor is the optimum duration of follow-up for these patients.

Current national guidelines provide evidence-based and comprehensive advice on treatment for DCIS and provide a framework for uniformity of management across all centres in the UK [2]. However, rapid advancements in the field of breast cancer treatment following the publication of this national guidance mean that it may not necessarily represent the contemporaneous views of front-line clinicians. Hence critical examination of the views and opinions of those responsible for managing patients with DCIS on a daily basis is vital to understanding the current paradigm of DCIS management. Furthermore, a cross-sectional survey of surgeons views towards modern DCIS treatment will provide a context when interpreting deviations in national audit and routinely collected data on treatment trends compared with those expected from strict adherence to national guidance.

We hypothesised that surgeons' attitudes towards the treatment of DCIS varied by seniority of surgeon and by geographical location. We aimed to (i) investigate whether there were correlations between seniority and geographical location of surgeon with attitudes towards DCIS terminology and treatment, respectively, (ii) determine the degree of variation between surgeons in the management of DCIS across the UK, (iii) understand the current paradigm of DCIS management amongst those who treat the condition and to document the potential scope of over- and undertreatment between regions.

## 2. Methodology

We conducted a mixed methods study involving qualitative interviewing and a semiquantitative survey over an eight-week period from 4th January 2015 until 1st March 2015. An online survey was constructed and hosted by an internet-based survey website. The survey was targeted primarily at breast surgeons treating women with DCIS. The anonymous survey contained questions regarding age, profession, seniority, and geographical location of the respondent in addition to specific questions regarding DCIS management, identified during a systematic search of the literature during survey development phase.

### 2.1. Survey Development

The survey was developed following a systematic review of the published literature. A systematic review was conducted using Medline/Pubmed, OVID/Embase, and Google scholar search engines. Areas of uncertainties and controversies in all aspects of DCIS were identified. These were coded and summarised into a list (Supplementary Appendix  1 in Supplementary Material available online at http://dx.doi.org/10.1155/2015/104231). Questions were then devised around these areas of uncertainty to produce a pilot survey.

### 2.2. Pilot Survey

A random sample of five independent breast surgeons completed the survey and their responses were analysed in addition to a process of cognitive interviewing that was undertaken regarding their thoughts, interpretation, and opinions regarding the strengths and limitations on each of the survey questions and the survey. The survey was subsequently modified in response to this cognitive interviewing and the revised survey underwent a repeated pilot phase by a further random sample of five different and independent breast surgeons until the survey was determined as satisfactory and the questions as objective as possible (Supplementary Appendix  2). Responses from the pilot and development phases of the study survey were not included in the final analysis.

### 2.3. Survey Dissemination and Recruitment

In order to obtain a nonbiased sample we aimed to disseminate the survey equally to all breast surgeons and breast trainees across the country. This was achieved via a nationwide breast surgery group who were contacted to cascade the survey equally to all of their respective members. A reminder email was sent fourteen days later to maximise response rate.

### 2.4. Statistical Analysis

Descriptive statistics and nonparametric tests were conducted comparing responses to questions by surgeon seniority and geographical location. For the purposes of the analysis, deaneries were grouped into three broad geographical categories; Scotland, North of England, and South of England.

## 3. Results

Overall 61.2% (*n* = 49/80) of responding surgeons completed the entire survey. The survey took respondents approximately ten minutes to complete and surgeons who did not complete the survey in full were not included in further analyses. Approximately 57% of respondents were male and 89.8% of participants were aged 30–49 years and either consultant (39%) or specialist registrar (ST3) (37%). Approximately 63% of participants were based in district general hospitals and 37% were based in university hospitals (Table 1). All training deaneries were represented and respondents undertook an average of 19 breast surgery cases per month.

### 3.1. Terminology Preferred Varies by Surgeon Seniority

The majority of respondents agreed with describing DCIS as “abnormal cells in the milk ducts” (57%) and “pre-cancer” (76%). There were increasing levels of heterogeneity in answers from respondents with increasing levels of seniority to terms such as tumour, neoplasm, and malignancy (Table 2). In contrast, when comparing terminology by respondents' monthly workload of cancer surgery, responses were largely similar between surgeons with higher (≥20 cases per month) and lower (<20 cases per month) caseloads. The exception to this was use of the terms “tumour” and “cancer,” which were more preferable to surgeons with higher caseloads than to those with lower caseloads (Supplementary Appendix  3). Geographically, approximately 54.6% and 72% of surgeons in North of England and in Scotland, respectively, disagreed with the term “tumour” compared to only 40% of surgeons in South of England. Similar results were seen for the term “malignancy.” There was no significant difference between the views of surgeons from district general hospitals and university hospitals both within and between regions in relation to this question.

### 3.2. Surgeons' Estimation of DCIS Prognosis Varies by Geographical Location

The majority of surgeons attributed a higher risk of progression to IBC or death with a higher pathological grade of DCIS. The majority (45%) of surgeons attributed the risk of low-grade DCIS progressing to invasive breast cancer at 10 years to be between 10 and 19%. 49% of surgeons felt that the risk of intermediate grade DCIS progressing to invasive breast cancer was 20–49%, and this risk rose to over 50% for high-grade (63%). Approximately 29% of surgeons in Scotland believed that high-grade DCIS had >50% risk of invasive cancer at 10 years compared to 80% of surgeons in South of England and 59% in North of England. There were no significant differences in surgeons' views in relation to the risk of mortality from DCIS between surgeons working at university or district general hospitals and between male and female surgeons. Overall the majority of surgeons estimated this risk for low-grade DCIS to be 1% (39%), for intermediate grade DCIS to be 2–5% (43%), and for high-grade DCIS to be more than 6% (61%). Surgeons from Scotland considered that there is a smaller risk of death across the three pathological grades when compared to their counterparts from England (Table 3); however there was little variation by individual surgeons' caseload (Supplementary Appendix  4).

### 3.3. Surgery for DCIS

The vast majority of respondents felt that breast-conserving surgery alone is adequate for large (4 cm) low-grade DCIS lesion. A higher proportion of female surgeons suggested mastectomy with sentinel lymph node biopsy (SLNB), as did a higher proportion of more junior surgeons. However, there were no statistically significant differences in these findings overall. The majority of surgeons advised a reexcision for women with a margin thickness of less than 0.5 mm. Approximately 57% of surgeons from Scotland advised a reexcision for 0.6–0.9 mm thickness compared to 96% and 85% in Northern and Southern England, respectively. There were no significant differences by gender or university or district general hospital. Regarding axillary surgery, the presence of a palpable lump (65%), a large lesion (>4 cm) (55%), or the presence of extensive microcalcification (27%) were the main indications for SLNB in DCIS listed by respondents. There was no significant variation in answers to this question by demographic or geographic factors (Table 4).

### 3.4. Adjuvant Treatment and Follow-Up for DCIS

Only 23% of respondents felt that premenopausal women who have had breast-conserving surgery with adequate margins for small low-grade DCIS require postoperative radiotherapy, in accordance with NICE guidelines. However, 71% did not agree with this and were equally represented across geographical regions and levels of seniority. However, approximately 65% of surgeons agreed with NICE guidance and felt that tamoxifen therapy was not required in this case. This proportion fell significantly when considering* high-grade* DCIS where only 40.8% of surgeons would withhold tamoxifen (Table 4).

The majority of surgeons (65%) felt that women with DCIS should be followed up for 5 years after treatment, 25% felt that <5 years is adequate, and 10% felt that follow-up longer than 5 years was required. These views did not vary by region, setting, or seniority. Approximately 25% of surgeons felt that annual mammograms with repeat referral if any abnormality is detected was the ideal follow-up for these women. An equal proportion (25%) felt that women should be seen by an oncologist for the first year and then annual mammograms thereafter. Approximately 20% felt that women should be seen regularly during this period by the breast surgeon and only 2% felt that input of both breast surgeon and oncologist was required for the entire 5 years.

## 4. Discussion

Although previous research has focused on women's understanding of different aspects of DCIS and its management [3–8], there has been little work investigating surgeons' views towards areas of uncertainty in DCIS [9]. We present the only survey to our knowledge that has focused on UK surgeons and specifically explored the qualitative opinions of breast surgeons towards various facets of DCIS management. Our findings confirm the hypothesis that the views of surgeons towards the prognosis and various aspects of management of DCIS vary geographically across the UK. In addition there are differences in terminology preferred by surgeons of varying seniority when communicating DCIS to their patients. Our findings also show that surgeons deviate from national guidance on areas such as indications for SLNB, tamoxifen, and follow-up practice and that they may prioritise contemporary evidence or personal experience when dealing with these areas of uncertainty.

The majority of respondents agreed with describing DCIS as “abnormal cells in the milk ducts” and “pre-cancer.” However, of interest in our study, there became increasing levels of heterogeneity in answers from respondents of varying levels of seniority to terms such as “tumour,” “neoplasm,” and “malignancy.” More senior surgeons disagreed with terms such as “cancer” or “neoplasm,” possibly due to increased awareness of the emotional association by patients of such terms with adverse outcomes. DCIS is generally treated differently from invasive breast cancer in the UK with the absence of SLNB following wide local excision and the absence of Tamoxifen therapy postoperatively [2]. Hence, more senior surgeons may feel that using these terms usually reserved for invasive breast cancer when counselling women with DCIS may only serve to increase confusion.

Whereas data from NSABP B-24 randomised trial showed the annual risk of invasive ipsilateral breast tumor recurrences after lumpectomy to be 0.88 events per 100 DCIS patients per year [10], respondents to our survey generally overestimated the perceived risk from DCIS (Table 3). This was also the case for the risk of death following a diagnosis of DCIS. Although all-cause mortality data following a diagnosis of DCIS is sparse, breast cancer specific mortality following DCIS has been analysed using the Surveillance, Epidemiology, and End Results (SEER) 18 registries database and the 10-year breast cancer–specific mortality rate is estimated to be 1.1% after a diagnosis of DCIS [11]. Our findings suggest that surgeons view the risk of DCIS to be proportional to the grade of DCIS with high-grade lesions having the poorest prognosis in the absence of treatment. Although national guidance does not reflect any differentiation on treatment based on pathological grade [2], surgeons appear to have a stratified approach to DCIS risk based on grade. Surgeons in Scotland viewed DCIS as having a lower risk of disease progression to invasive cancer or to mortality at 10 years when compared to their counterparts in England. They also accepted a smaller margin thickness following surgical excision than their counterparts in southern deaneries. Vacuum assisted biopsy is performed more often in Scotland than in England but it is unclear whether the reported reduced underestimation rate from this biopsy procedure may influence more liberal opinions towards acceptable margin size.

Our findings also show that the views of surgeons deviated from national guidance on areas such as SLNB, tamoxifen, and follow-up in DCIS. NICE guidelines advise against the routine use of SLNB in patients with a preoperative diagnosis of DCIS who are having breast-conserving surgery, unless there is palpable mass or extensive microcalcifications, which may increase their risk of underlying invasive cancer. Overall surgeons agreed with these guidelines; however some felt that a large lesion (>5 cm) should also be an indication for SLNB and this opinion appeared to be more apparent in respondents from England than from Scotland (Supplementary Appendix  5). Furthermore NICE guidelines also advise against the routine use of adjuvant Tamoxifen in women who have had breast-conserving surgery with adequate margins [2]. Many surgeons' responses were in compliance with NICE guidance on this facet of management; however a large proportion of surgeons responded that they would also advise against this guidance in certain settings where more recent evidence from randomised trials may take precedence. This has important implications for the interpretation of long-term outcome trends from nationally collected data, since assumptions on treatments received over time based on national guidance cannot be made without considering the degree to which variation from national guidance may occur (as shown in this survey).

It is clear that little consensus exists regarding how best to describe DCIS or its natural history [12]. This in turn can allow the potential for wide discrepancies in its management and result in inadvertent over- or undertreatment of this disease [13]. Efforts to understand these discrepancies and standardise management may improve patient care [14]. In this study we have attempted to determine the contemporary views of surgeons towards different facets of DCIS management to understand the current paradigm of DCIS treatment and, in turn, provide a platform upon which the clinical utility of further research in the field can be related. 

*Limitations*. We were limited by the low response rate (25%) to this survey; however, this is commonly expected with this type of study design [15]. Selection bias amongst respondents is an inherent source of potential confounding amongst all surveys. However it was reassuring to see an equal gender and age mix amongst respondents and to have all deaneries represented equally across the UK suggesting that there was no significant systemic bias in response. Future work is required to determine how the views of surgeons within the UK compared with counterparts in other European countries and in the developed and undeveloped World. Such work would help to understand the sociocultural context of DCIS management and provide a framework on which to discuss how to improve DCIS treatment worldwide.

## 5. Conclusion

It is clear that the management of DCIS continues to be a source of contention. Our survey reaffirms that, irrespective of national guidelines and attempts at uniformity, there continues to be a wide variety of views amongst breast surgeons regarding the natural history and optimal management of DCIS. By quantifying this variation, it is possible to take it into account when examining long-term outcome data from recurrences and all-cause mortality following treatment for DCIS. The next step is to expand this study further and to compare responses internationally, both between countries in the European Union and Worldwide.

## Supplementary Material

Following systematic literature review of the published literature over the last 10 years, all relevant papers were coded into categories based on the identified areas of uncertainty in the management of DCIS. The salient research areas that were identified are shown in Supplementary online appendix 1. A process of cognitive interviewing finalised the optimal question phrasing and survey structure. A sample of the final survey distributed to surgeons is shown in supplementary online appendix 2. More detailed analyses of responses to the survey by monthly workload (number of operations undertaken by the respondent per month) are shown in supplementary online appendices 3 and 4. Supplementary online appendix 5 shows a tabulation of how differences in seniority and region influence concordance of respondents' survey answers with NICE guidelines regarding the role of SLNB in DCIS.

## Figures and Tables

**Table 1:** Characteristics of Respondents who completed the survey in full.

		*N*	%
Age	21–29	2	4.1%
	30–39	25	51.0%
	40–49	19	38.8%
	50–59	3	6.1%

Sex	Female	21	42.9%
	Male	28	57.1%

Seniority	Junior	**20**	**40.8%**
	Core Trainee (CT1-2)/Senior House Officer (SHO)	1	2.0%
	Specialist Trainee (ST3+)	18	36.7%
	Trust grade	1	2.0%
	Senior	**29**	**59.2%**
	Associate Specialist	7	14.3%
	Post-Certificate of Completion of Training (CCT) Fellow	3	6.1%
	Consultant	19	38.8%

Setting	District general hospital	31	63.3%
	University hospital	18	36.7%

Region	North	**22**	**44.9%**
	East Midlands	3	6.1%
	Northeast	2	4.1%
	Northwest	9	18.4%
	West Midlands	3	6.1%
	Yorkshire and the Humber	5	10.2%
	South	**20**	**40.8%**
	East of England	4	8.2%
	Kent, Surrey, and Sussex	3	6.1%
	London LETB	5	10.2%
	Southwest	2	4.1%
	Thames Valley and Oxford	3	6.1%
	Wessex	3	6.1%
	Scotland	**7**	**14.3%**
	East of Scotland	6	12.2%
	West of Scotland	1	2.0%

Cases per month	<20	25	51.0%
	≥20	24	49.0%
	Mean (SD)	19.3	11.9
	Median (Range)	18	(0–60)

**Table 2:** Seniority and terminology (percentage in each group (*n*)).

		Strongly disagree	Disagree	Neither agree or disagree	Agree	Strongly agree
Abnormal cells in the milk ducts	Junior	10 (2)	10 (2)	5 (1)	65 (13)	10 (2)
	Senior	21 (3)	36 (5)	56 (8)	46 (6)	42 (7)
	Total	**10 (5)**	**14 (7)**	**18 (9)**	**39 (19)**	**18 (9)**

Pre-cancer	Junior	5 (1)	15 (3)	0	40 (8)	40 (8)
	Senior	15 (2)	31 (4)	20 (2)	57 (9)	77 (12)
	Total	**6 (3)**	**14 (7)**	**4 (2)**	**35 (17)**	**41 (20)**

Cancer	Junior	15 (3)	20 (4)	20 (4)	30 (6)	15 (3)
	Senior	35 (4)	52 (9)	11 (2)	76 (10)	26 (4)
	Total	**14 (7)**	**27 (13)**	**12 (6)**	**33 (16)**	**14 (7)**

Tumour	Junior	15 (3)	25 (5)	25 (5)	30 (6)	5 (1)
	Senior	46 (6)	67 (11)	26 (4)	51 (7)	10 (1)
	Total	**18 (9)**	**33 (16)**	**18 (9)**	**27 (13)**	**4 (2)**

Neoplasm	Junior	15 (3)	25 (5)	25 (5)	20 (4)	15 (3)
	Senior	36 (5)	73 (12)	36 (5)	41 (5)	15 (2)
	Total	**16 (8)**	**35 (17)**	**20 (10)**	**18 (9)**	**10 (5)**

Malignancy	Junior	15 (3)	25 (5)	30 (6)	20 (4)	10 (2)
	Senior	46 (6)	56 (8)	47 (8)	31 (4)	21 (3)
	Total	**18 (9)**	**27 (13)**	**29 (14)**	**16 (8)**	**10 (5)**

**Table 3:** Geographical differences in perceived prognosis for each DCIS grade (percentage in each group (*n*)).

DCIS Grade	Region	Risk of developing invasive Ca				Risk of death			
		1–9%	10–19%	20–49%	50%+	0%	1%	2–5%	6%+
Low	North	9 (2)	45 (10)	32 (7)	14 (3)	23 (5)	27 (6)	32 (7)	18 (4)
	South	30 (6)	40 (8)	20 (4)	10 (2)		40 (8)	45 (9)	15 (3)
	Scotland	43 (3)	57 (4)			14 (1)	71 (5)	14 (1)	
	Total	**22 (11)**	**45 (22)**	**22 (11)**	**10 (5)**	**12 (6)**	**39 (19)**	**35 (17)**	**14 (7)**

Intermediate	North	9 (2)	14 (3)	59 (13)	18 (4)	23 (5)	9 (2)	32 (7)	36 (8)
	South	5 (1)	25 (5)	40 (8)	30 (6)		10 (2)	55 (11)	35 (7)
	Scotland	29 (2)	29 (2)	43 (3)			43 (3)	43 (3)	14 (1)
	Total	**10 (5)**	**20 (10)**	**49 (24)**	**20 (10)**	**10 (5)**	**14 (7)**	**43 (21)**	**33 (16)**

High	North	5 (1)	5 (1)	32 (7)	59 (13)		5 (1)	27 (6)	68 (15)
	South	5 (1)		15 (3)	80 (16)			30 (6)	70 (14)
	Scotland		14 (1)	57 (4)	29 (2)		14 (1)	71 (5)	14 (1)
	Total	**4 (2)**	**4 (2)**	**29 (14)**	**63 (31)**		**4 (2)**	**35 (17)**	**61 (30)**

**Table 4:** Treatment and factors to advise sentinel lymph node biopsy and their agreement with guidelines (percentage in each group (*n*)). ^*∗*^NICE guideline 2009 (early breast cancer) [2].

		Disagree	Neither disagree nor agree	Agree	% responses agree with guideline^*∗*^
Treatment	Use of radiotherapy in low-grade DCIS	22 (11)	6 (3)	71 (35)	22
	Use of tamoxifen in low-grade DCIS	65 (32)	20 (10)	14 (7)	65
	Use of tamoxifen in high-grade DCiS	41 (20)	12 (6)	47 (23)	41

Factors to advise sentinel lymph node biopsy	Palpable lump	35 (17)		65 (32)	65
	Large lesion (>5 cm)	45 (22)		55 (27)	45
	Family history of invasive breast cancer	94 (46)		6 (3)	94
	Extensive microcalcifications on mammogram	73 (36)		27 (13)	27
	Nipple discharge	96 (47)		4 (2)	96
