# Supplementary material for: A Nationwide Cross-Sectional Survey of UK Breast Surgeons' Views on the Management of Ductal Carcinoma In Situ
Source: Int J Breast Cancer. 2015 Nov 30;2015:104231. doi: 10.1155/2015/104231 (PMC4677188; doi:10.1155/2015/104231)
Supplement: Supplementary file 1 — Following systematic literature review of the published literature over the last 10 years, all relevant papers were coded into categories based on the identified areas of uncertainty in the management of DCIS. The salient research areas that were identified are shown in Supplementary online appendix 1. A process of cognitive interviewing finalised the optimal question phrasing and survey structure. A sample of the final survey distributed to surgeons is shown in supplementary online appendix 2. More detailed analyses of responses to the survey by monthly workload (number of operations undertaken by the respondent per month) are shown in supplementary online appendices 3 and 4. Supplementary online appendix 5 shows a tabulation of how differences in seniority and region influence concordance of respondents' survey answers with NICE guidelines regarding the role of SLNB in DCIS. [file 104231.f1.pdf]

Supplementary online appendix 1:

Identification of survey questions:

Following systematic literature review of the published literature over the last 10 years, all relevant papers were coded into categories based on which areas of uncertainty in the management of DCIS they identified as important. The salient research questions identified were as follows:

Criteria:
1. Published in the last 10 years (2005-2015)
2. Published in the English language.
3. Clear description and justification of area of uncertainty in the management of DCIS
4. Clear recommendation for further research

Areas of uncertainty identified, upon which survey questions were developed:
i. Definition and nomenclature of DCIS (53 papers)
ii. The natural history of DCIS (12 papers)
iii. Theories regarding disease progression (30 papers)
iv. Uncertainties in risk factors for DCIS (11 papers)
v. Developing prognostic indices for women with DCIS and the VNPI (9 papers)
vi. Uncertainties in Breast Surgery for DCIS (36 papers)
vii. Controversies regarding Core-Biopsy in DCIS: (9 papers)
viii. Uncertainties in axillary staging (42 papers)
ix. Uncertainties in adjuvant radiotherapy (30 papers)
x. Uncertainties in adjuvant hormonal therapy (17 papers)
xi. Uncertainties regarding the Need for surveillance (8 papers)
xii. Evolving incidence of DCIS (4 papers)

Supplementary online appendix 2: Survey questions and structure.

Ductal Carcinoma in Situ (DCIS) survey

Welcome to the DCIS Survey

Thank you for participating in this quick 5 minute survey.

Your help is very important to us.

This is an anonymous survey and no identifiable information will be collected or shared.

Please click next to begin.

Ductal Carcinoma in Situ (DCIS) survey

About you

This is an anonymous survey and no identifiable information will be collected or shared.

* 1. Are you male or female?

- Male
- Female

* 2. What is your age?

- 17 or younger
- 18-20
- 21-29
- 30-39
- 40-49
- 50-59
- 60 or older

* 3. Please select your level of seniority:

- Consultant
- Associate Specialist
- Post-Certificate of Completion of Training (CCT) Fellow
- Trust grade
- Specialist Trainee (ST3+)
- Core Trainee (CT1-2) / Senior House Officer (SHO)
- Surgical Nurse Practitioner

Other, please specify

* 4. Do you work in a district general hospital or a university-affiliated hospital?

- University Hospital
- District General Hospital

* 5. In what region of the UK do you work?

- ◯ **East Midlands**
  (Nottinghamshire, Derbyshire, Lincolnshire, Leicester, Kettering and Northampton)
- ◯ **East of England**
  (Cambridgeshire, Norfolk, Suffolk, Bedfordshire, Hertfordshire and Essex)
- ◯ **Kent, Surrey & Sussex**
- ◯ **London Local Education Training Boards (LETBs)**
  (Health Education North Central and East London, Health Education North West London and Health Education South London)
- ◯ **North East**
  (Newcastle upon Tyne, North Cumbria, Cumberland, Gateshead, North Tees, Hartlepool, Durham, Darlington, Sunderland, South Tees, Northumbria, Wansbeck and North Tyneside)
- ◯ **North West**
  (previously called Mersey and North Western Deanery) (Aintree, Alder Hey, East Cheshire, Liverpool, Mid Cheshire, The Isle of Mann, Southport, Walton, Warrington, Manchester, Stockport, Tameside, Pennine, Blackpool, Bolton, Lancashire, Salford, Morecambe Bay, Wigan, Wrightington and Leigh)
- ◯ **East of Scotland**
  (Edinburgh, Fife, Perth, Dundee, Aberdeen, Inverness, Elgin, Fort William and Stornaway)
- ◯ **West of Scotland**
  (Greater Glasgow & Clyde, Lanarkshire, Ayrshire & Arran, Forth Valley, Dumfries And Galloway)
- ◯ **South West**
  (previously called Severn and South West Peninsula Deanery) (Cheltenham, Gloucester, Swindon, Bristol, Bath, Weston & Taunton, Peninsula, Devon, Cornwall, Gloucestershire, Cheltenham, Bristol, Southmead Hospital, Bath, Swindon, Taunton & Somerset)
- ◯ **Thames Valley and Oxford**
  (Oxfordshire, Berkshire and Buckinghamshire, Banbury, Milton Keynes, Wexham Park, Reading, Slough, High Wycombe and Aylesbury)
- ◯ **Wales**
  (Swansea, Bridgend, Newport, Rhyl,Bangor, Wrexham, Cardiff, Merthyr Tydfil, Llantrisant, Carmarthen, Haverfordwest)
- ◯ **Wessex**
  (Hampshire, Dorset, Isle of Wight and Salisbury, Jersey and Guernsey)
- ◯ **West Midlands**
  (Birmingham & Black Country, Shropshire & Staffordshire, Warwickshire & Worcestershire)
- ◯ **Yorkshire and the Humber**
  (Airedale, Barnsley, Bradford, Calderdale and Huddersfield (Halifax), Chesterfield, Doncaster, Bassetlaw, Harrogate, Hull, Leeds, Mid Yorkshire Hospitals NHS Trust, Wakefield, Grimsby, Scunthorpe, Rotherham, Sheffield, York)
- ◯ Other (please specify)

* 6. Approximately how many breast cases do you operate on (or assist with) per month? (please enter a number)

Ductal Carcinoma in Situ (DCIS) survey

Terminology and Prognosis of DCIS

* 7. How strongly do you agree with the following terms for describing DCIS to patients:

	Strongly Disagree	Disagree	Neither Disagree Nor Agree	Agree	Strongly Agree
Abnormal cells in the milk ducts	◯	◯	◯	◯	◯
Pre-cancer	◯	◯	◯	◯	◯
Cancer	◯	◯	◯	◯	◯
Tumour	◯	◯	◯	◯	◯
Neoplasm	◯	◯	◯	◯	◯
Maligancy	◯	◯	◯	◯	◯

* 8. A 50 year old patient is diagnosed with screening detected 5mm **low grade** DCIS. What would you estimate her percentage (%) risk without any treatment of:

(i) developing invasive cancer at 10 years to be: (Please enter a whole number between 1-100) [ ]

(iii) death at 10 years to be: (Please enter a whole number between 1-100) [ ]

* 9. A 50 year old patient is diagnosed with screening detected 5mm **intermediate grade** DCIS. What would you estimate her percentage (%) risk without any treatment of:

(i) developing invasive cancer at 10 years to be: (Please enter a whole number between 1-100) [ ]

(iii) death at 10 years to be: (Please enter a whole number between 1-100) [ ]

* 10. A 50 year old patient is diagnosed with screening detected 5mm **high grade** DCIS. What would you estimate her percentage (%) risk without any treatment of:

(i) developing invasive cancer at 10 years to be: (Please enter a whole number between 1-100) [ ]

(iii) death at 10 years to be: (Please enter a whole number between 1-100) [ ]

Ductal Carcinoma in Situ (DCIS) survey

Surgery for DCIS

* 11. A pre-menopausal woman with 4cm low grade, unifocal DCIS with limited microcalcification in C-cup (average size) breast asks which surgical option you would advise. Please select one from the list below:

* 12. A premenopausal woman with 10mm intermediate grade DCIS has a wire guided wide local excision from skin to muscle. Would you advise a re-excision of margin for the following margin thickness:

	Yes	No
2.1 - 10.0 mm:	○	○
1.0 - 2.0 mm:	○	○
0.6 - 0.9 mm:	○	○
0.2 - 0.5 mm:	○	○
0.1 mm:	○	○
0mm (with involved margin):	○	○

* 13. Which method does your unit most often use to obtain a pre-operative sample of radiologically detected DCIS for histological analysis:

- ○ Vacuum-assisted biopsy
- ○ 14G Core needle biopsy
- ○ Fine-needle aspiration
- ○ Don't know

* 14. 2. A 50yr old women presents with a screening detected, biopsy proven DCIS breast lesion. She is to be treated with breast conserving surgery. Would any of these factors make you also advise sentinel lymph node biopsy in this patient:

	Yes	No
Palpable lump	○	○
Large lesion (>5cm)	○	○
Family history of invasive breast cancer	○	○
Extensive microcalcifications on mammogram	○	○
Nipple discharge	○	○

Ductal Carcinoma in Situ (DCIS) survey

Adjuvant treatment and follow up for DCIS

* 15. Premenopausal women who had breast conserving surgery for a **6 mm low grade** DCIS with a 2 mm margin thickness does not require postoperative radiotherapy:

Strongly Disagree	Disagree	Neither Disagree Nor Agree	Agree	Strongly Agree
○	○	○	○	○

* 16. Premenopausal women who had breast conserving surgery for a **6 mm low grade** DCIS with a 2 mm margin thickness, should be treated with post-operative tamoxifen therapy for at least 5 years following her primary surgery and treatment:

Strongly Disagree	Disagree	Neither Disagree Nor Agree	Agree	Strongly Agree
○	○	○	○	○

* 17. Premenopausal women with **20 mm high grade DCIS** and 2 mm margin thickness, should be treated with post-operative tamoxifen therapy for at least 5 years following her primary surgery and treatment:

Strongly Disagree	Disagree	Neither Disagree Nor Agree	Agree	Strongly Agree
○	○	○	○	○

* 18. A premenopausal woman with 10mm DCIS has wire guided wide local excision with 2mm nearest margin with post-operative radiotherapy.

How many years should this woman be followed up post-operatively (enter whole number):

[ ]

How many times should she be seen in total within this period (e.g. if once a year for 5 years, then the answer is 5 times, if twice a year for 10 years, then the answer is 20 times) (enter whole number):

[ ]

* 19. Regarding the patient in the previous question above (question: 18); who should follow her up:

[ ]

Other (please specify)

[ ]

Ductal Carcinoma in Situ (DCIS) survey

Thank you

Thank you very much for taking the time to complete this survey on DCIS.

You have made an important contribution and it is very much appreciated.

Please be assured that no identifiable information is collected and all responses are anonymous.

Please click the Done tab to exit.

Supplementary online appendix 3: Cases and Terminology (percentage in each group (n)).

	Number of Cases	Strongly Disagree	Disagree	Neither Disagree Nor Agree	Agree	Strongly Agree
**Abnormal cells in the milk ducts**	< 20	4 (1)	12 (3)	8 (2)	44 (11)	32 (8)
	>= 20	17 (4)	17 (4)	29 (7)	33 (8)	4 (1)
	**Total**	**10 (5)**	**14 (7)**	**18 (9)**	**39 (19)**	**18 (9)**
**Pre-cancer**	< 20	12 (3)	12 (3)	4 (1)	36 (9)	36 (9)
	>= 20	0 (0)	17 (4)	4 (1)	33 (8)	46 (11)
	**Total**	**6 (3)**	**14 (7)**	**4 (2)**	**35 (17)**	**41 (20)**
**Cancer**	< 20	16 (4)	28 (7)	12 (3)	24 (6)	20 (5)
	>= 20	13 (3)	25 (6)	13 (3)	42 (10)	8 (2)
	**Total**	**14 (7)**	**27 (13)**	**12 (6)**	**33 (16)**	**14 (7)**
**Tumour**	< 20	16 (4)	40 (10)	24 (6)	16 (4)	4 (1)
	>= 20	21 (5)	25 (6)	13 (3)	38 (9)	4 (1)
	**Total**	**18 (9)**	**33 (16)**	**18 (9)**	**27 (13)**	**4 (2)**
**Neoplasm**	< 20	12 (3)	32 (8)	28 (7)	16 (4)	12 (3)
	>= 20	21 (5)	38 (9)	13 (3)	21 (5)	8 (2)
	**Total**	**16 (8)**	**35 (17)**	**20 (10)**	**18 (9)**	**10 (5)**
**Malignancy**	< 20	16 (4)	24 (6)	36 (9)	12 (3)	12 (3)
	>= 20	21 (5)	29 (7)	21 (5)	21 (5)	8 (2)
	**Total**	**18 (9)**	**27 (13)**	**29 (14)**	**16 (8)**	**10 (5)**

Supplementary online appendix 4: Number of cases for each DCIS grade (percentage in each group (n)).

DCIS Grade	Number of Cases	Risk of Developing Invasive Ca				Risk of Death			
		1-9%	10-19%	20-49%	50%+	0%	1%	2-5%	6%+
Low	< 20	28 (7)	40 (10)	16 (4)	16 (4)	12 (3)	44 (11)	24 (6)	20 (5)
	>= 20	17 (4)	50 (12)	29 (7)	4 (1)	13 (3)	33 (8)	46 (11)	8 (2)
	**Total**	**22 (11)**	**45 (22)**	**22 (11)**	**10 (5)**	**12 (6)**	**39 (19)**	**35 (17)**	**14 (7)**
Intermediate	< 20	12 (3)	16 (4)	52 (13)	20 (5)	8 (2)	24 (6)	48 (12)	20 (5)
	>= 20	8 (2)	25 (6)	46 (11)	21 (5)	13 (3)	4 (1)	38 (9)	46 (11)
	**Total**	**10 (5)**	**20 (10)**	**49 (24)**	**20 (10)**	**10 (5)**	**14 (7)**	**43 (21)**	**33 (16)**
High	< 20	4 (1)	4 (1)	40 (10)	52 (13)		4 (1)	48 (12)	48 (12)
	>= 20	4 (1)	4 (1)	17 (4)	75 (18)		4 (1)	21 (5)	75 (18)
	**Total**	**4 (2)**	**4 (2)**	**29 (14)**	**63 (31)**		**4 (2)**	**35 (17)**	**61 (30)**

Supplementary online appendix 5: How differences in seniority and Region influence concordance with NICE guidelines regarding the role of SLNB in DCIS (overall percentage of positive answers (n)). *NICE guideline 2009 (Early breast cancer) (2).

		Palpable lump		Large lesion		Family history		Extensive Micro-calcifications		Nipple discharge	
		**Agree**	**Disagree**	**Agree**	**Disagree**	**Agree**	**Disagree**	**Agree**	**Disagree**	**Agree**	**Disagree**
Overall N(%) agree with NICE guidance*		65 (32)	35 (17)	45 (22)	55 (27)	94 (46)	6 (3)	27 (13)	73 (36)	96 (47)	4 (2)
**Seniority**	Junior	70 (14)	30 (6)	50 (10)	50 (10)	95 (19)	5 (1)	35 (7)	65 (13)	95 (19)	5 (1)
	Middle Grade	60 (6)	40 (4)	30 (3)	70 (7)	90 (9)	10 (1)	30 (3)	70 (7)	100 (10)	-
	Senior	63 (12)	37 (7)	47 (9)	53 (10)	95 (18)	5 (1)	16 (3)	84 (16)	95 (18)	5 (1)
**Region**	North	68 (15)	32 (7)	41 (9)	59 (13)	91 (20)	9 (2)	36 (8)	64 (14)	100 (22)	-
	Scotland	43 (3)	57 (4)	71 (5)	29 (2)	100 (7)	-	-	100 (7)	86 (6)	14 (1)
	South	70 (14)	30 (6)	40 (8)	60 (12)	95 (19)	5 (1)	25 (5)	75 (15)	95 (19)	5 (1)
